# MADSP: predicting anti-cancer drug synergy through multi-source integration and attention-based representation learning

**DOI:** 10.1093/bioinformatics/btaf326

**Published:** 2025-06-03

**Authors:** Yuqi Hong, Qichang Zhao, Jianxin Wang

**Affiliations:** Hunan Provincial Key Lab on Bioinformatics, School of Computer Science and Engineering, Central South University, Changsha 410083, China; Hunan Provincial Key Lab on Bioinformatics, School of Computer Science and Engineering, Central South University, Changsha 410083, China; Hunan Provincial Key Lab on Bioinformatics, School of Computer Science and Engineering, Central South University, Changsha 410083, China

## Abstract

**Motivation:**

Drug combination therapy is an effective strategy for cancer treatment, enhancing drug efficacy and reducing toxic side effects. However, *in vitro* drug screening experiments are time-consuming and expensive, necessitating the development of computational methods for drug synergy prediction. While current methods focus on molecular chemical structures, they often overlook the biological context, limiting their ability to capture complex drug synergies.

**Results:**

In this work, we propose MADSP, a novel method for anti-cancer drug synergy prediction that integrates target and pathway knowledge for a more comprehensive understanding of systems biology. MADSP first incorporates chemical structure, target, and pathway features of drugs, using a multi-head self-attention mechanism to learn a unified drug representation. It then integrates protein–protein interaction data with omics data from cell lines, extracting a low-dimensional dense embedding of cell lines via an autoencoder. Finally, the synergy scores for drug combinations are predicted using a fully connected neural network. Experiments on benchmark datasets demonstrate that MADSP outperforms state-of-the-art methods. The ablation study reveals that multi-source information fusion and attention mechanisms significantly enhance model performance. The case study further illustrates the practical applicability of MADSP as a powerful tool for drug synergy prediction, offering potential for advancing cancer treatment strategies.

**Availability and implementation:**

MADSP is available at https://github.com/Hhyqi/MADSP.

## 1 Introduction

Human diseases are complex and ever-changing, involving multiple factors such as biological mechanisms, genetic factors, and environmental influences (Hunter 2005). This complexity is not only reflected in the causes and progression of diseases but also in various symptoms and personalized treatments. Compared with traditional monotherapy, the use of two or more drugs, namely combination therapy (Mokhtari *et al.* 2017), can intervene in the development of diseases from multiple aspects. For diseases such as cancer (Al-Lazikani *et al.* 2012), combination therapy can produce synergistic effects, improve the treatment effect on tumors (Jia *et al.* 2009), and reduce the side effects of monotherapy (Tooker *et al.* 2007, Huang *et al.* 2017). However, drug combinations may also lead to antagonistic impacts, reduce the therapeutic effect of drugs, and even in some cases, trigger toxicity, endangering the life of patients (Hecht *et al.* 2009). To identify which combinations exhibit synergistic effects, pharmaceutical researchers often employ high-throughput screening (HTS) (Bajorath 2002) techniques to test the effects of multiple drug combinations (He *et al.* 2018). The enormous size of drug combinations presents a major challenge to HTS (Jiménez-Luna *et al.* 2020). Therefore, reliable computational methods are needed to identify and evaluate drug combinations more efficiently (Tsigelny 2019).

Early computational studies are inspired by systems biology and established statistical models (Nelander *et al.* 2008, Feala *et al.* 2010). However, these methods struggled to simulate complex nonlinear problems. With the rapid development of artificial intelligence methods, several machine learning-based have been proposed to improve prediction accuracy. For instance, Celebi *et al.* (2019) utilized multi-omics data and predicted potential synergistic drug combinations by XGBoost. Gayvert *et al.* (2017) used random forests as the classifier to predict high-throughput drug screening data. Furthermore, using the molecular fingerprints of the drugs, the concentration values of the drugs, and the gene expression profiles of the cancer cell lines as inputs, Julkunen *et al.* (2020) proposed a machine-learning framework called comboFM. comboFM applies a higher-order factorization machine to learn higher-order features of drug pairs for the prediction of drug combinations in preclinical studies based on cell lines or patient-derived cells.

In recent years, the emergence of publicly available large-scale datasets centered on drug discovery (O’Neil *et al.* 2016) provides data to support subsequent research (Zhao *et al.* 2022, 2025). Deep learning methods are becoming increasingly popular in drug combination prediction due to their superior predictive performance and wide range of predictive capabilities. For instance, DeepSynergy (Preuer *et al.* 2018) took molecular structural fingerprints and genomic features of cell lines as input, and employed a multi-layer perceptron to obtain the final prediction results. Matchmaker (Kuru *et al.* 2022) input the drug chemical descriptors and a cell line gene expression profile as features to predict the Loewe score. The main contribution of Matchmaker is its ability to learn a representation of each drug conditioned on cell line gene expression. In addition to the above methods, several models use protein–protein interactions (PPIs) and drug–drug interactions as supplementary information to enhance prediction performance. For instance, PRODeepSyn (Wang *et al.* 2022) used graph convolutional networks to integrate PPI networks with omics data to construct low-dimensional dense embeddings for cell lines. SNRMPACDC (Li *et al.* 2023) processed the features of two drugs using a Siamese network and introduced a random matrix projection module to get the interaction between drugs. PermuteDDS (permutedds) designed a Permutable feature fusion mechanism to combine drug and cell line features to capture the complex relationship between drug combinations and cell lines. HypertranSynergy (wang2024granularity) used hypergraphs to express multivariate relationships between cancer cell lines and drug combinations, and predicted anti-cancer drug synergistic effects based on a granular-level information fusion strategy with hypergraph transformers.

While the above methods have their advantages by improving the characterization of drugs and cell lines, they rely solely on the chemical structure of the drug and relatively sparse training data, making the characterization of drugs and cell lines inadequate. Moreover, the existing methods ignore the rich knowledge contained in drug databases and the linkages between drugs and other biological entities, which are crucial for exploring the effects of drug combinations. Increasing evidence suggests that drug response might be regulated by the cooperative behavior of multiple genes rather than by individual genes alone (Shi *et al.* 2017). The introduction of pathway information helps to account for this coordination among genes, reducing model complexity and enhancing the interpretability of predictive models (Khatri *et al.* 2012). By acting on multiple targets simultaneously, drugs can overcome cancer cell resistance and improve therapeutic efficacy (Knight *et al.* 2010). Drug pathways and target information can provide detailed mechanisms of drug action within the cell, including how they affect specific biological signaling pathways, metabolic pathways, and protein interaction networks. By integrating this knowledge, synergistic effects between drugs can be predicted more comprehensively and accurately. Another challenge is that structural descriptors of drugs and genomic features of cell lines are very sparse. Extracting dense drug and cell line embeddings is key to further improving drug synergy prediction.

To this end, we propose MADSP, a deep-learning method for anti-cancer drug synergy. MADSP gathers multiple features of drugs to introduce the context of the biological properties, including chemical structure, targets, and pathways, and integrate these features with a multi-head self-attention mechanism to learn cross-modal drug representations. MADSP integrate PPIs with cell line omics data and use an autoencoder to extract low-dimensional dense embeddings. Based on the embeddings of drugs and cell lines, the model predicts the synergy scores for drug combinations using the fully connected neural networks (FCNNs). The experiments indicate that the performance of MADSP is superior to state-of-the-art baselines. We also conduct independent testing experiments to demonstrate the predictive ability of MADSP for new drugs and new cell lines. Moreover, we conduct ablation experiments to demonstrate the effectiveness of each feature and the validity of each component. Finally, we conducted case studies to assess the consistency of our model with research results.

## 2 Materials and methods

**Figure 1.**
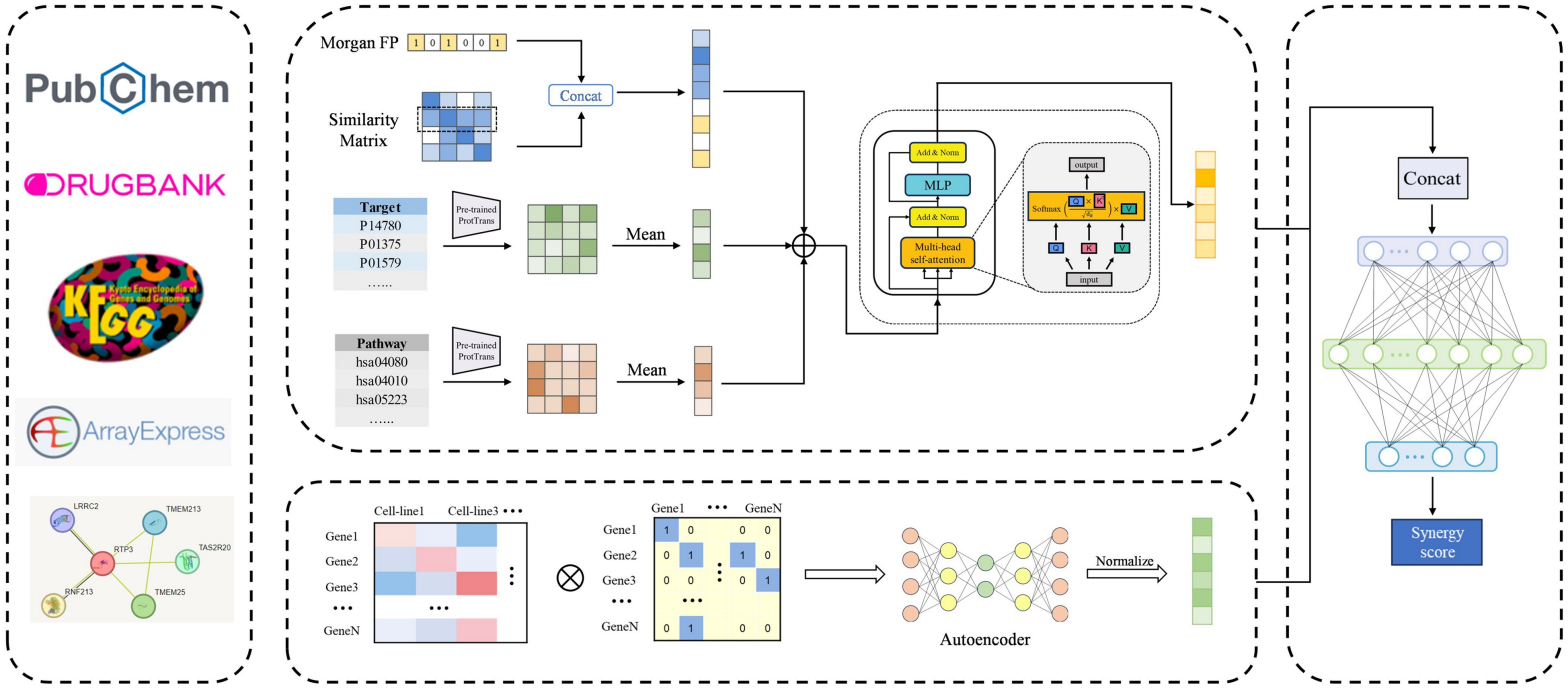
Overview of MADSP.

The proposed MADSP consists of three parts, as shown in Fig. 1. (i) Drug feature extraction module. MADSP uses SMILES string, pathway, and target information as input and learn the ensemble feature representation of drug combinations using a multi-head attention mechanism. The multi-head attention mechanism enables the model to focus on different parts of the input data, thus effectively capturing potential dependencies. (ii) Cell line feature extraction module. For cell lines, MADSP fuses PPI network with genomic features of cell lines and extracts low-dimensional dense embeddings using an autoencoder. The autoencoder extract low-dimensional and high-level feature representations, which reduces the interference of input noise. (iii) Prediction module. Drug and cell line features are concatenated and processed by a three-layer FCNN with dropout.

### 2.1 Benchmarks

For regression task, we use the O’Neil dataset published by Merck *et al.* (O’Neil *et al.* 2016) as the benchmark. The dataset covers 583 different combinations constructed from 38 different anti-cancer drugs and 39 human cancer cell lines. Each experimental sample consists of two drugs and one cell line, which contains 22 737 samples. To assess the ability to predict novel drug combinations, we use the 5-fold cross-validation, in which the datasets are divided into five disjoint folds, where the same drug combinations would only appear in the same fold. The synergistic effect of the drug combination is represented using four types: Loewe (Greco *et al.* 1995), Bliss (Bliss 1939), ZIP (Yadav *et al.* 2015), and HSA (Berenbaum 1989), respectively. Higher synergy scores typically indicate stronger combination synergies.

For the classification task, we use the Loewe score on the O’Neil dataset and set the threshold to 30 to obtain categorical labels. Furthermore, we perform classification tasks on the DrugCombDB dataset (Liu *et al.* 2021). DrugCombDB contains 448 555 drug–drug combinations, covering 2887 unique drugs and 124 human cancer cell lines. As we require drug structure and cell line gene expression as features, we screen these data so that drugs have structural information available from the Drugbank database and cell lines have gene expression data available from the CCLE database. At the same time, there are some replicates in the original data. For each triplet, we average the collaborative scores of the replicates. The final reduced dataset consists of 69 436 samples, with 764 unique drugs and 76 unique cell lines. For the DrugCombDB dataset, synergy scores are expressed as ZIP values. Since the synergy score is a continuous value, we set the threshold to 0 to separate the data sample into synergy and antagonism.

### 2.2 Drug feature generation

As shown in Fig. 1, we collect three types of features for drugs, including the chemical structure, the target, and pathway knowledge. For the chemical structure features, we utilize rdkit (Landrum *et al.* 2013) to generate morgan fingerprints (Morgan 1965) for each drug based on their SMILES strings, with a radius of 3 and a length of 1024 dimensions. The morgan fingerprints are encoded as 1 or 0 to indicate the presence or absence of the corresponding substructure. Since the morgan fingerprint is a high-dimensional sparse vector, we use the similarity to represent the structure feature of drugs to reduce the sparsity. The jaccard similarity is calculated as follows:

(1) $$J(d_{i},d_{j})=\frac{\left|d_{i}^{mf}\cap d_{j}^{mf}\right|}{\left|d_{i}^{mf}\cup d_{j}^{mf}\right|}=\frac{\left|d_{i}^{mf}\cap d_{j}^{mf}\right|}{\left|d_{i}^{mf}|+|d_{j}^{mf}|-|d_{i}^{mf}\cap d_{j}^{mf}\right|},$$

where drugs $d_{i}^{mf}$ and $d_{j}^{mf}$ are the morgan fingerprints of drugs $d_{i}$ and $d_{j}$, respectively. After similarity calculation, we get a low-dimensional dense embedding $d_{i}^{sm}=[J(d_{i},d_{1}),\ldots ,J(d_{i},d_{38})]\in R^{38}$ for drug $d_{i}$.

Given that drug synergism is usually achieved by inhibiting discrete biological targets (Jin *et al.* 2021), we retrieve known target interactions from the DrugBank database (Knox *et al.* 2011). We use the drug IDs from the PubChem database (Li *et al.* 2010) to search for drug information in the DrugBank database. This process allows us to obtain information about the drug targets. After screening and integration, we obtain a total of 112 target proteins. We use ProtTrans (Julkunen *et al.* 2020) as the feature extraction of protein sequences, which is based on the Transformer architecture. Through pre-training on a large amount of protein sequence data, ProtTrans can capture complex patterns and relationships within protein sequences. Each drug’s feature based on targets is the mean of the corresponding target feature vectors:

(2) $$d^{t}=\frac{1}{n}\sum_{i=1}^{n}T_{i},$$

where $T_{i}\in R^{1024}$ represents the feature vector of the *i*-th target involved in the drug, and *n* is the total number of targets involved in the drug. By averaging these target feature vectors, we obtain the composite feature $d_{i}^{t}\in R^{1024}$ for each drug.

To further introduce the biological context of the drugs, we retrieved the pathway tags of the drugs from the KEGG database (Kanehisa *et al.* 2010). Pathway tags as abstract biological concepts involve multiple genes. Therefore, we use sequence features of genes to characterize pathway information. Specifically, Pathway tag $P_{i}$ involves *m* genes, i.e. $\left[\mathrm{gen}e_{1},\mathrm{gen}e_{2},\ldots ,\mathrm{gen}e_{m}\right]$, the features of Pathway tag $P_{i}$ are constructed in the following way:

(3) $$P_{i}=\frac{1}{m}\sum_{j=1}^{m}Pr\mathrm{otTrans}(G^{j}),$$

where $G^{j}$ is the amino acid sequence of $\mathrm{gen}e_{j}$.

For drug $d_{i}$ related to *k* pathway tags, we get a pathway feature $d_{i}^{p}\in R^{1024}$.

(4) $$d_{i}^{p}=\frac{1}{k}\sum_{j=1}^{k}P_{j}.$$

Finally, for each drug, we pad the three features from the different sources mentioned above into a unified dimensional by zero-padding. For a pair of i-th drug and j-th drug, we get a combination feature $d_{ij}=\{d_{i}^{mf},d_{i}^{sm},d_{i}^{t},d_{i}^{p},d_{j}^{mf},d_{j}^{sm},d_{j}^{t},d_{j}^{p}\}\in R^{8*d}.$

Considering the complexity of drug synergy mechanisms, we use a multilayer transformer structure to model potential interactions between drugs. Taking the combination feature $d_{ij}$ as input, we obtain representations for the query, key, and value of the combination of drugs, $Q=W_{q}(d_{ij})$, $K=W_{k}(d_{ij})$, $V=W_{v}(d_{ij})$. The output of the self-attention layer is calculated by matrix operation as follows:

(5) $$\text{Attention}(Q,K,V)=\text{Softmax}\left(\frac{QK^{T}}{\sqrt{d}}\right)V,$$

where $1/\sqrt{d}$ is the scaling factor to prevent the problem of vanishing gradient. To get the semantic information in different spaces, for each layer of the transformer structure, we use a multi-head attention mechanism. For the j-th layer, the output $O_{j}$ is generated as follows:

(6) $${\;\text{head}\;}_{i}=\text{Attention}\;\left(Q_{i},K_{i},V_{i}\right),$$

 

(7) $$O_{j}=\text{Concat}\;({\text{head}}_{1},\ldots ,{\text{head}}_{h})W^{O},$$

where *h* denotes the number of heads. For better optimization of the model, residual connection and layer normalization are used. The final output $O\in R^{8\times d}$ of the drug combination can be obtained by learning from the multilayer transformer structure. To apply *O* to the prediction module, we flatten it into a vector $f_{d}\in R^{8d}$:

(8) $$f_{d}=\text{FLATTEN}\left(O\right).$$

### 2.3 Cell line feature generation

High-dimensional and sparse omics data are often employed for feature construction in cell lines. Considering the importance of interactions between proteins based on gene expression in drug combination therapy (Li *et al.* 2018), we utilize the omics features of cell lines and protein correlation matrices to construct cell line features. Cell line gene expression data are obtained from the ArrayExpress database (Iorio *et al.* 2016) and normalized. Cell line gene mutation data are collected from the COSMIC cell line project (Tate *et al.* 2019).

The interactions between gene expression products can be collected from the PPI networks generated by PRODeepSyn (Wang *et al.* 2022). This PPI network is extracted from the STRING database (Szklarczyk *et al.* 2019) and PPIs with a composite score lower than 0.7 are excluded, resulting in a total of 839 522 interactions involving 17 161 proteins. These proteins are translated to genes through the protein-gene mapping file provided by the STRING database to be associated with gene expression and gene mutation data.

Note that the gene expression matrix of the cell line is $C_{e}\in R^{N_{\mathrm{cell}}\times M_{e}}$, the gene mutation matrix of the cell line is $C_{\mathrm{mut}}\in R^{N_{\mathrm{cell}}\times M_{\mathrm{mut}}}$, where $N_{\mathrm{cell}}$ is the number of cell lines, and *M* is the embedding dimension. The protein association matrix is $C_{\mathrm{ppi}}\in R^{t\times t}$, and $t\in \left\{M_{e},M_{\mathrm{mut}}\right\}$ is the matrix dimension. The feature fusion matrix of the cell line $C\in R^{N_{\mathrm{cell}}\times \left(M_{\mathrm{mut}}+M_{e}\right)}$ can be expressed as:

(9) $$C=\text{Concat}\left(C_{\mathrm{mut}}⊗C_{\mathrm{ppi}},C_{e}⊗C_{\mathrm{ppi}}\right),$$

where ⊗ is the tensor product.

Since the dimensionality of the cell line feature data is very large, we use a deep autocoder as a dimensionality reduction tool for the cell line features in our model. Autoencoder (Baldi 2012) is a typical unsupervised deep learning model that is capable of reconstructing low-dimensional, sparse, and noiseless inputs subject to a number of constraints. Autoencoder can be considered as a composition of the encoder and decoder functions represented as follows:

(10) $$h_{i}=f_{\theta }(C_{i}),$$

 

(11) $$C_{i}^{\prime }=g_{\theta^{\prime }}(h_{i})=g_{\theta^{\prime }}(f_{\theta }(C_{i})).$$

The reconstruction loss $L(C_{i},C_{i}^{\prime })$ between the original input vector $C_{i}$ and the reconstructed vector $C_{i}^{\prime }$ is utilized Backpropagation to guide the training process and update the parameters θ and $\theta^{\prime }$ that reduce the reconstruction loss.

(12) $$L\;\left(C_{i},C_{i}^{\prime }\right)=\sum_{i=1}^{N}||C_{i}-g_{\theta^{\prime }}\left(f_{\theta }(C_{i})\right)|{|}_{2}.$$

The autoencoder encodes the feature vector $C_{i}$ obtained from the feature matrix *C* into a hidden representation $h_{i}$, and then reconstructs it using a decoder. For the cell line *c*, the final feature is downscaled to a vector $f_{c}=f_{\theta }(c)\in R^{768}$.

### 2.4 Predictive modules

Taking the drug combination feature $f_{d}$ and the low-density embedding of cell line $f_{c}$, we construct a three-layer neural network to predict the drug synergy scores.

(13) $${\hat{y}}_{i}=\text{DNN}\left(f_{d},f_{c}\right).$$

We use rectified linear units as the activation function. Between the layers, we add a batch normalization layer to accelerate convergence and a dropout layer to enhance the generalization of the model.

We adopt mean squared error (MSE) as the loss function for regression task. The MSE is defined as follows:

(14) $$\mathrm{Los}s_{\mathrm{mse}}(y_{i},{\hat{y}}_{i})=\frac{1}{N}\sum_{i=1}^{N}{\left(y_{i}-{\hat{y}}_{i}\right)}^{2},$$

where $y_{i}$ represents the true value of the i-th sample and ${\hat{y}}_{i}$ represents the predicted value.

We trained our model using the pytorch (Paszke *et al.* 2019) framework. Using an early-stop strategy, training is automatically stopped if no improvement is observed within 20 cycles to prevent model overfitting. We set the batch size to 512 and dropout rate to 0.5, and used Adam with default parameters as the optimization algorithm to train the network. We set *h* to 8 and *d* to 1024 for multi-head self-attention mechanism.

## 3 Results

### 3.1 Experimental setup

We conduct performance comparisons between MADSP and baselines through 5-fold cross-validation. More details on the baselines are provided in the Section S1, available as supplementary data at *Bioinformatics* online. To assess the ability of the methods to predict novel drug combinations, we employ the sample partitioning method outlined by Preuer *et al.* (2018). All data are divided into five non-overlapping folds, ensuring that the same drug combinations appeared only in the same fold. The final results are obtained by averaging the validation results from each fold. All baselines use the same data partitioning with original hyperparameter setting.

We choose four evaluation metrics that have been widely used to provide a comprehensive assessment of the regression performance, the MSE, Pearson correlation coefficient (PCC), Spearman correlation coefficient (SCC), and consistency index (CI). The classification experiments utilize the following metrics: Area Under the ROC Curve (AUC), Accuracy (ACC), Precision, and Area Under the PR Curve (AUPR).

### 3.2 Performance evaluation

**Figure 2.**
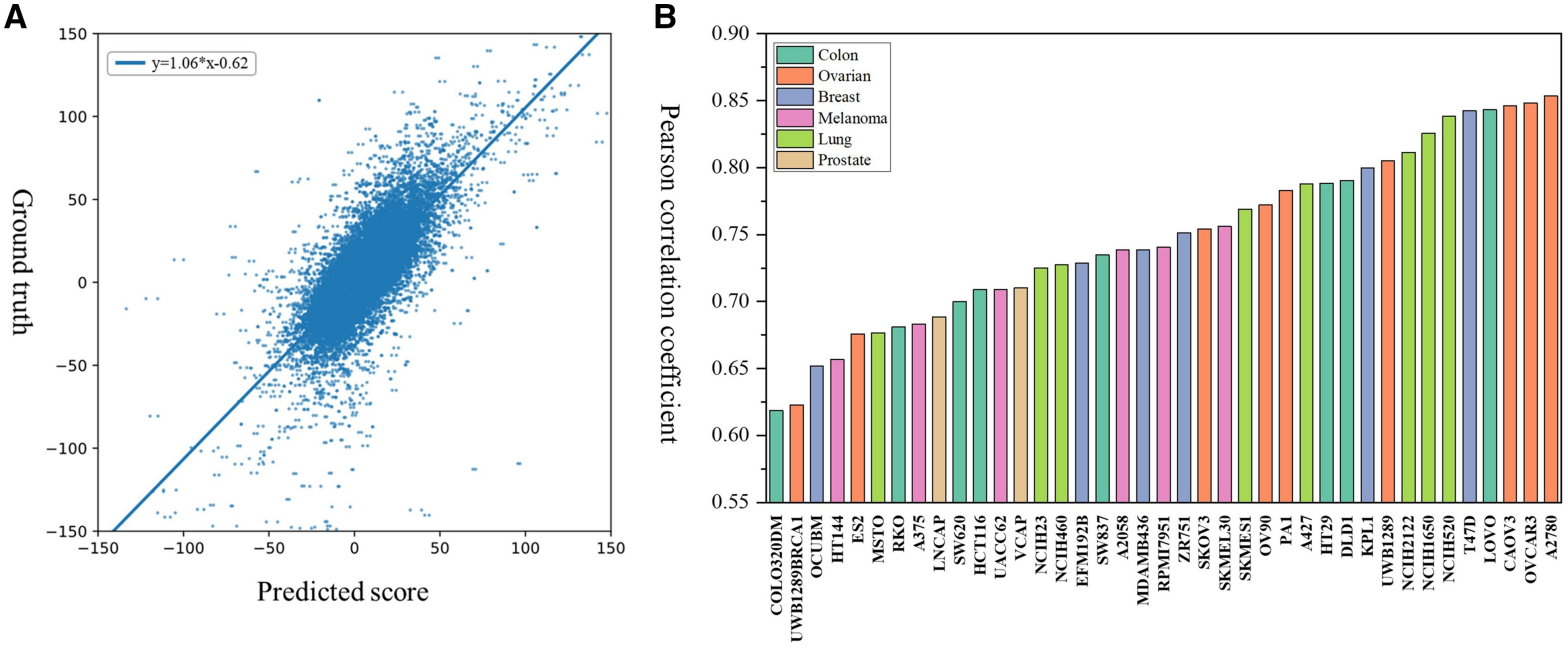
The correlation of the model predictions with labels. (A) Scatter plot of predicted scores and true values. The blue line is a plot of the function fitted by least squares regression with a slope of 1.06 and a deviation of 0.62. (B) PCC values for each cell line. The colors of the bars represent the tissue of the cell lines.

We evaluate the performance of MADSP in predicting the synergistic scores of drug combinations across all cell lines. Figure 2A shows the correlation between the predictions and the true values of all data points. Most of the points are clustered around the fitted line and the PCC reaches 0.77. The PCC and the fitted function of the MADSP indicate a strong correlation between the predicted results and the actual values. We summarize the PCC between the predicted scores and the true values for each cell line according to the tissue type is shown in Fig. 2B, and the color of the bars shows the tissue to which the cell line belongs. COLOR320DM had the lowest PCC of 0.62 and A2780 had the highest PCC of 0.87. Only 2 of the 39 cell lines had a PCC below 0.65, while 19 had a PCC above 0.75. From the results, the predictions of MADSP exhibit a strong correlation with the ground truth.

There is no significant association between the PCC values and the types of tissues, indicating that MADSP holds potential value in predicting synergistic anti-cancer drug combinations for different tissue cell lines.

We compare MADSP with state-of-the-art methods by 5-fold cross-validation. Table 1 summarizes the performance of different methods under four synergy types on the benchmark dataset. As shown in Table 1, MADSP achieves the lowest MSE for Loewe, Bliss, ZIP, and HSA synergy types, with values of 216.24, 23.87, 24.51, and 16.48, respectively. MADSP also attains the highest PCC, SCC, and CI value across all synergy types. The results indicate that MADSP outperforms other methods across all four synergy types. The introduction of pathway and target features can provide connections between drugs and other biological entities, enhancing the model’s understanding of drug synergy. Moreover, MADSP can capture the interaction patterns between features through multi-head attention mechanisms, further improving predictive performance.

**Table 1.** Performance of different methods on benchmark datasets of four synergy types.

Method	MSE	PCC	SCC
Loewe			
SVR	423.01 (56.09)	0.44 (0.02)	0.50 (0.02)
Random-Forest	325.39 (47.02)	0.62 (0.02)	0.63 (0.03)
DeepSynergy	277.23 (43.67)	0.69 (0.03)	0.68 (0.02)
Matchmaker	254.75 (46.48)	0.72 (0.03)	0.72 (0.03)
PermuteDDS	241.80 (44.26)	0.73 (0.03)	0.71 (0.02)
SNRMPACDC	235.59 (44.14)	0.73 (0.02)	0.72 (0.02)
HypertranSynergy	233.82 (42.17)	0.73 (0.03)	0.70 (0.02)
PRODeepSyn	229.79 (35.42)	0.75 (0.02)	0.74 (0.02)
**MADSP**	**216.24** (**43.37**)	**0.77** (**0.02**)	**0.75** (**0.02**)
Bliss			
SVR	44.68 (5.14)	0.46 (0.03)	0.53 (0.02)
Random Forest	33.08 (4.69)	0.65 (0.03)	0.70 (0.02)
DeepSynergy	37.24 (5.32)	0.60 (0.02)	0.63 (0.02)
PermuteDDS	35.38 (7.85)	0.62 (0.07)	0.71 (0.03)
HypertranSynergy	34.15 (8.53)	0.64 (0.06)	0.68 (0.02)
Matchmaker	29.43 (3.59)	0.70 (0.02)	0.74 (0.01)
SNRMPACDC	26.36 (3.63)	0.73 (0.03)	0.74 (0.02)
PRODeepSyn	25.83 (3.17)	0.74 (0.02)	**0.76** (**0.02**)
**MADSP**	**23.87** (**3.47**)	**0.75** (**0.02**)	**0.76** (**0.01**)
HSA			
SVR	44.98 (4.28)	0.45 (0.02)	0.53 (0.01)
Random-Forest	36.67 (3.72)	0.59 (0.03)	0.65 (0.03)
Matchmaker	34.97 (3.91)	0.68 (0.02)	0.71 (0.02)
DeepSynergy	35.87 (3.98)	0.61 (0.02)	0.66 (0.01)
HypertranSynergy	34.68 (9.61)	0.63 (0.07)	0.68 (0.03)
PermuteDDS	34.21 (9.51)	0.64 (0.07)	0.70 (0.03)
SNRMPACDC	27.32 (3.54)	0.72 (0.02)	0.73 (0.02)
PRODeepSyn	26.30 (2.99)	0.73 (0.02)	0.74 (0.01)
**MADSP**	**24.51** (**3.01**)	**0.74** (**0.02**)	**0.75** (**0.02**)
ZIP			
SVR	30.22 (4.07)	0.48 (0.02)	0.50 (0.02)
DeepSynergy	25.11 (3.75)	0.61 (0.02)	0.61 (0.01)
Random Forest	21.74 (3.29)	0.67 (0.02)	0.68 (0.01)
HypertranSynergy	20.90 (2.71)	0.68 (0.03)	0.67 (0.02)
Matchmaker	19.61 (3.01)	0.73 (0.01)	0.75 (0.01)
PermuteDDS	19.38 (2.43)	0.70 (0.02)	0.70 (0.03)
SNRMPACDC	17.31 (3.17)	0.75 (0.02)	0.75 (0.02)
PRODeepSyn	17.18 (4.13)	0.75 (0.01)	0.76 (0.01)
**MADSP**	**16.48** (**4.04**)	**0.76** (**0.02**)	**0.76** (**0.01**)

Bold values highlight the best performance values.

Considering that previous study (Preuer *et al.* 2018) treats the drug combination prediction as a classification task, we also modify MADSP into a classification model and conduct related experiments on the O’Neil dataset. Table 2 reports the classification performance of the different methods on the benchmark dataset using Loewe scores. MADSP performs the best AUPR with a value of 0.63. AUPR is effective in reflecting the model’s ability to predict imbalanced samples. Additionally, MADSP demonstrates good performance in AUC, ACC, and Precision, with values of 0.91, 0.94, and 0.73, respectively. Therefore, MADSP exhibits strong competitiveness in classification tasks as well.

**Table 2.** Comparison of the results of MADSP with baselines on the O’Neil dataset for the classification task.

Method	AUPR	AUC	ACC	Precision
SVR	0.45 (0.11)	0.78 (0.03)	0.89 (0.02)	0.70 (0.18)
Random Forest	0.53 (0.03)	0.85 (0.03)	0.93 (0.01)	0.65 (0.03)
DeepSynergy	0.55 (0.04)	0.87 (0.04)	0.92 (0.01)	0.59 (0.07)
Matchmaker	0.57 (0.05)	0.89 (0.02)	0.93 (0.01)	0.72 (0.03)
PermuteDDS	0.58 (0.05)	0.89 (0.02)	0.93 (0.01)	0.70 (0.07)
SNRMPACDC	0.60 (0.03)	0.90 (0.02)	**0.94** (**0.01**)	**0.73** (**0.02**)
PRODeepSyn	0.62 (0.04)	0.90 (0.02)	0.91 (0.01)	0.69 (0.05)
HypertranSynergy	0.62 (0.03)	0.90 (0.02)	0.93 (0.02)	0.72 (0.04)
**MADSP**	**0.63** (**0.03**)	**0.91** (**0.02**)	**0.94** (**0.01**)	**0.73** (**0.03**)

Bold values highlight the best performance values.

To further investigate the generalization performance of the model on different datasets, we compare it with the classification model GraRep (Cao *et al.* 2015), DeepSynergy, GraphSynergy (Yang *et al.* 2021), NEXGB (Meng *et al.* 2022), and DeepTraSynergy (Rafiei *et al.* 2023) on the DrugCombDB dataset. Detailed results are provided in Table S3, available as supplementary data at *Bioinformatics* online. The existing methods are difficult to achieve other drugs that do not exist in the current dataset. However, by using pre-trained methods to characterize targets and pathways and introducing Morgan fingerprints of drugs, MADSP can generalize better than existing models.

### 3.3 Ablation study

To further investigate the impact of each drug feature on the performance of MADSP, we construct variants of the MADSP model with different feature combinations. Since the chemical structure features of drugs are the most essential feature of the drug molecule, we retain the chemical structure feature of drugs in each feature combination during the ablation experiments. The variant model *Mpt* ignores target and pathway information and only utilizes the chemical structure for prediction. The variant model *Mt* uses the structure and pathway information of drugs while ignoring target features for prediction. The variant model *Mp* excludes pathway features and takes the structural features and targets of drugs as inputs. The prediction results are shown in Table 3. Compared with only using the structure feature, drug feature combinations significantly improve the predictive performance of the models. Furthermore, the combination of drug chemical structure, target, and pathway features performs the best results. The results indicate that the introduction of multi-source data can enable the model to obtain more information and achieve better predictive performance.

**Table 3.** Performance evaluation of ablation studies on MADSP.

Method	MSE	PCC	SCC
Mpt	227.33 (42.74)	0.76 (0.02)	0.73 (0.02)
Mt	225.53 (43.71)	0.76 (0.03)	0.74 (0.02)
Mp	223.88 (38.54)	0.76 (0.02)	0.74 (0.01)
Mppi	226.89 (40.65)	0.76 (0.03)	0.74 (0.02)
Matt	228.01 (43.99)	0.75 (0.02)	0.75 (0.01)
**MADSP**	**216.24** (**43.37**)	**0.77** (**0.02**)	**0.75** (**0.02**)

Bold values highlight the best performance values.

To verify the impact of the PPI matrix and the attention mechanism module on the model performance, we construct two variants of MADSP:

- **Mppi**: The model removes the PPI matrix and standardize the concatenated gene expression and mutation features of the cell lines.
- **Matt**: The model removes the attention mechanism module and directly combines the features of the drug as input to the prediction module.

As shown in Table 3, the MSE decreases significantly after removing the PPI matrix or the attention mechanism. The protein interaction matrix contains the interaction relationship between cell line genes and these interactions contain the molecular mechanisms of drug action and help develop personalized medicine strategies to optimize drug selection and dosage. In addition, the attention mechanism helps the target and Pathway features to automatically adjust the drug pair features, which can further enhance the model performance.

### 3.4 Independent test

We further use the leave-one-out cross-validation to comprehensively evaluate the generalization performance of our methods. Table 4 summarizes the independent test results of MADSP on the benchmark dataset using Loewe scores. We devise two strategies for cold-start experiments: the leave-one-drug-out strategy considers all drug combinations containing a particular drug as test samples, while the leave-one-cell-line-out strategy treats each cell line in turn as the test object.

**Table 4.** Comparison of model performance of leave-one-out cross-validation.

Method	RMSE	PCC	SCC	CI
Leave-one-cell-line-out				
SVR	20.40	0.57	0.59	0.71
Random Forest	19.93	0.59	0.60	0.72
DeepSynergy	20.50	0.56	0.58	0.71
Matchmaker	20.75	0.56	0.57	0.71
SNRMPACDC	19.71	0.58	0.59	0.72
PRODeepSyn	19.53	0.58	0.60	0.72
PermuteDDS	19.36	0.53	0.55	0.70
HypertranSynergy	18.65	0.59	0.60	0.72
**MADSP**	**18.01**	**0.59**	**0.61**	**0.72**
Leave-one-drug-out				
SVR	21.55	0.35	0.39	0.64
Random Forest	20.86	0.47	0.47	0.67
DeepSynergy	20.81	0.48	0.47	0.67
Matchmaker	20.52	0.48	0.48	0.67
PermuteDDS	20.42	0.45	0.49	0.67
SNRMPACDC	20.27	0.47	0.42	0.65
PRODeepSyn	19.66	0.49	0.48	0.67
HypertranSynergy	19.16	0.46	0.45	0.66
**MADSP**	**18.95**	**0.52**	**0.50**	**0.68**

Bold values highlight the best performance values.

The predictive performance of all methods is relatively low when generalized to new drugs and cell lines. MADSP performs best in all four metrics for both leave-one-drug-out and leave-one-cell-line-out. Under the leave-one-drug-out strategy, the test drug did not appear in the training set. Through structural similarity, MADSP can infer the potential characteristics of new drugs, which combined with the drug’s target and Pathway characteristics ensures the predictive ability in the leave-one-drug-out setting. This cross-drug knowledge transfer can compensate for the lack of new drug data. Independent testing results indicate the stability of MADSP when applied to new drugs or cell lines.

### 3.5 Case study

To further investigate the effectiveness of MADSP in predicting new synergistic drug combinations, we conduct a case study on the benchmark dataset to validate whether MADSP can predict novel synergistic drug combinations. We predict 4524 sample data for 116 drug combinations that do not appear in the training set. We then focus on the top 100 sample combinations with the highest prediction scores, and conduct a relevant literature search. We find that seven predicted drug combinations are consistent with the observed results of previous studies or clinical trial observations, as shown in Table 5. The threshold for predicting synergistic effects is 30, and drug combinations above this value have synergistic effects.

**Table 5.** Case study on new synergistic drug combinations.

Cancer type	Drug A	Drug B	Cell line	Prediction	Rank
Lung	Paclitaxel	Dasatinib	MSTO	71.09	9
Ovarian	Carboplatin	Dasatinib	OVCAR3	65.45	13
Ovarian	Carboplatin	Dasatinib	A2780	55.71	25
Lung	Paclitaxel	Dasatinib	A427	50.41	31
Lung	Paclitaxel	Dasatinib	NCIH520	42.03	56
Ovarian	Carboplatin	AZD1775	UWB1289	37.75	78
Ovarian	Carboplatin	AZD1775	UWB1289BRCA1	37.58	80

In combination with Dasatinib and Paclitaxel (Diao *et al.* 2016), Dasatinib promotes necrotic apoptosis in Paclitaxel-treated lung adenocarcinoma cells. Both Dasatinib and Carboplatin showed significant inhibitory effects on the ovarian cancer cell line OVCAR3, and the combination of the two drugs had a synergistic effect. AZD1775 can make TP53 mutant ovarian cancer cells more sensitive to platinum-based chemotherapeutic agents such as Carboplatin (Laing *et al.* 2016).

Experiments illustrate that the combination of SORAFENIB and DASATINIB has a synergistic effect on melanoma cell line A2058, and inhibition of BRAF and KIT, the corresponding targets of SORAFENIB, reduces melanoma cell proliferation (Quattrini *et al.* 2019, Wang *et al.* 2021). MAPK/ERK, the pathway corresponding to DASATINIB, can regulate the MAPK signaling pathway by inducing apoptosis and inhibiting cell invasion (Guan and Huang 2022). We removed BRAF, KIT, and MAPK/ERK features during model inference, resulting in a decrease in the predicted value of [SORAFENIB, DASATINIB, A2058] from 35.5 to 31.7 (labeled 36.3).

The drug combination SUNITINIB and LAPATINIB acted synergistically against the human ovarian cancer cell line SKOV3, modulating the PI3K/AKT/mTOR pathway corresponding to SUNITINIB and inhibiting HER2 target proteins corresponding to LAPATINIB, which inhibited SKOV3 cell proliferation and tumor growth (Komarova *et al.* 2011, Yang *et al.* 2017). Our removal of HER2 and PI3K/AKT/mTOR features during model inference resulted in a decrease in the predicted value of [SUNITINIB, LAPATINIB, SKOV3] from 30.2 to 27.2 (labeled 31.2).

Overall, the case study results highlight the effectiveness of MADSP in accurately predicting and revealing drug combinations in different cellular environments, providing valuable insights into drug synergism and resistance mechanisms.

## 4 Conclusion

Drug combination therapy is a complex biological process involving numerous intricate interactions, many of which remain unknown. Developing accurate predictive models of drug synergy can significantly aid in drug discovery. Previous research has primarily focused on drug and cell line characterization, often neglecting the integration of drug-related knowledge, which limits the applicability and generalizability of these models. In this study, we propose MADSP, a novel computational approach that integrates multi-source data from drugs and cell lines to predict drug synergy. MADSP utilizes a self-attention mechanism to effectively fuse multi-source drug features and incorporates PPI information into cell line representations.

Our experimental results show that MADSP outperforms existing state-of-the-art methods in predicting drug combination synergies. Additionally, independent testing and case studies demonstrate the model’s capability to predict novel drug–cell line interactions and drug combinations with high accuracy. These findings highlight the promising potential of MADSP as a powerful tool for advancing drug synergy prediction and facilitating clinical applications in precision medicine.

Despite these achievements, MADSP has some limitations. While drug target and pathway information are crucial, other factors, such as pharmacodynamics and pharmacokinetics, also play a significant role in drug synergy prediction. Genetic polymorphisms affecting drug metabolism, transporters, and drug–drug interactions are critical for understanding how drugs interact in the body and tailoring personalized therapies. To enhance the accuracy and interpretability of drug synergy predictions, future research should focus on constructing a comprehensive pharmacokinetic and pharmacodynamic knowledge graph. This could form the foundation for developing more advanced models that integrate biological knowledge and provide actionable insights for improving drug synergy predictions.

## Supplementary Material

btaf326_Supplementary_Data

## Data Availability

The data underlying this article are available in https://github.com/Hhyqi/MADSP.

## References

[btaf326-B1] Al-Lazikani B , Banerji U, Workman P et al Combinatorial drug therapy for cancer in the post-genomic era. Nat Biotechnol 2012;30:679–92. 22781697 10.1038/nbt.2284

[btaf326-B2] Bajorath J. Integration of virtual and high-throughput screening. Nat Rev Drug Discov 2002;1:882–94. 12415248 10.1038/nrd941

[btaf326-B3] Baldi P. Autoencoders, unsupervised learning, and deep architectures. In: *Proceedings of ICML Workshop on Unsupervised and Transfer Learning*. JMLR Workshop and Conference Proceedings, Bellevue, Washington, USA, 2012, 37–49.

[btaf326-B4] Berenbaum MC. What is synergy? Pharmacol Rev 1989;41:93–141. 2692037

[btaf326-B5] Bliss CI. The toxicity of poisons applied jointly 1. Ann Appl Biol 1939;26:585–615.

[btaf326-B7] Cao S, Wei L, Qiongkai X et al GraRep: learning graph representations with global structural information. In: *Proceedings of the 24th ACM International on Conference on Information and Knowledge Management*. 2015, Melbourne, VIC, Australia.

[btaf326-B8] Celebi R , Bear Don’t Walk O, Movva R et al In-silico prediction of synergistic anti-cancer drug combinations using multi-omics data. Sci Rep 2019;9:8949. 31222109 10.1038/s41598-019-45236-6 PMC6586895

[btaf326-B10] Diao Y , Ma X, Min W et al Dasatinib promotes paclitaxel-induced necroptosis in lung adenocarcinoma with phosphorylated caspase-8 by c-Src. Cancer Lett 2016;379:12–23. 27195913 10.1016/j.canlet.2016.05.003

[btaf326-B11] Feala JD , Cortes J, Duxbury PM et al Systems approaches and algorithms for discovery of combinatorial therapies. Wiley Interdiscip Rev Syst Biol Med 2010;2:181–93. 20836021 10.1002/wsbm.51

[btaf326-B12] Gayvert KM , Aly O, Platt J et al A computational approach for identifying synergistic drug combinations. PLoS Comput Biol 2017;13:e1005308. 28085880 10.1371/journal.pcbi.1005308 PMC5234777

[btaf326-B13] Greco WR , Bravo G, Parsons JC. The search for synergy: a critical review from a response surface perspective. Pharmacol Rev 1995;47:331–85. 7568331

[btaf326-B14] Guan J , Huang H. Chrysophanol induces cell apoptosis and suppresses cell invasion by regulating AKT and MAPK signaling pathway in melanoma cells. ScienceAsia 2022;48:558.

[btaf326-B15] He L , Kulesskiy E, Saarela J et al Methods for high-throughput drug combination screening and synergy scoring. Methods Mol Biol 2018;1711:351–98. 29344898 10.1007/978-1-4939-7493-1_17 PMC6383747

[btaf326-B16] Hecht JR , Mitchell E, Chidiac T et al A randomized phase IIIb trial of chemotherapy, bevacizumab, and panitumumab compared with chemotherapy and bevacizumab alone for metastatic colorectal cancer. J Clin Oncol 2009;27:672–80. 19114685 10.1200/JCO.2008.19.8135

[btaf326-B17] Huang Y , Jiang D, Sui M et al Fulvestrant reverses doxorubicin resistance in multidrug-resistant breast cell lines independent of estrogen receptor expression. Oncol Rep 2017;37:705–12. 28000875 10.3892/or.2016.5315 PMC5355712

[btaf326-B18] Hunter DJ. Gene–environment interactions in human diseases. Nat Rev Genet 2005;6:287–98. 15803198 10.1038/nrg1578

[btaf326-B19] Iorio F , Knijnenburg TA, Vis DJ et al A landscape of pharmacogenomic interactions in cancer. Cell 2016;166:740–54. 27397505 10.1016/j.cell.2016.06.017 PMC4967469

[btaf326-B20] Jia J , Zhu F, Ma X et al Mechanisms of drug combinations: interaction and network perspectives. Nat Rev Drug Discov 2009;8:111–28. 19180105 10.1038/nrd2683

[btaf326-B21] Jiménez-Luna J , Grisoni F, Schneider G et al Drug discovery with explainable artificial intelligence. Nat Mach Intell 2020;2:573–84.

[btaf326-B22] Jin W , Stokes JM, Eastman RT et al Deep learning identifies synergistic drug combinations for treating COVID-19. Proc Natl Acad Sci USA 2021;118:e2105070118. 34526388 10.1073/pnas.2105070118 PMC8488647

[btaf326-B23] Julkunen H , Cichonska A, Gautam P et al Leveraging multi-way interactions for systematic prediction of pre-clinical drug combination effects. Nat Commun 2020;11:6136. 33262326 10.1038/s41467-020-19950-z PMC7708835

[btaf326-B24] Kanehisa M , Goto S, Furumichi M et al Kegg for representation and analysis of molecular networks involving diseases and drugs. Nucleic Acids Res 2010;38:D355–60. 19880382 10.1093/nar/gkp896 PMC2808910

[btaf326-B25] Khatri P , Sirota M, Butte AJ et al Ten years of pathway analysis: current approaches and outstanding challenges. PLoS Comput Biol 2012;8:e1002375. 22383865 10.1371/journal.pcbi.1002375 PMC3285573

[btaf326-B26] Knight ZA , Lin H, Shokat KM et al Targeting the cancer kinome through polypharmacology. Nat Rev Cancer 2010;10:130–7. 20094047 10.1038/nrc2787 PMC2880454

[btaf326-B27] Knox C , Law V, Jewison T et al DrugBank 3.0: a comprehensive resource for ‘omics’ research on drugs. Nucleic Acids Res 2011;39:D1035–41. 21059682 10.1093/nar/gkq1126 PMC3013709

[btaf326-B28] Komarova TV , Kosorukov VS, Frolova OY et al Plant-made trastuzumab (herceptin) inhibits HER2/Neu+ cell proliferation and retards tumor growth. PLoS One 2011;6:e17541. 21390232 10.1371/journal.pone.0017541 PMC3048398

[btaf326-B29] Kuru HI , Tastan O, Cicek AE et al MatchMaker: a deep learning framework for drug synergy prediction. IEEE/ACM Trans Comput Biol Bioinform 2022;19:2334–44. 34086576 10.1109/TCBB.2021.3086702

[btaf326-B30] Laing N , Lai Z, Barrett JC et al Genetic analysis of tumors from a phase II trial evaluating AZD1775, carboplatin and paclitaxel in patients with TP53-mutant ovarian cancer. Cancer Res 2016;76:337.

[btaf326-B31] Landrum G et al RDKit: Cheminformatics and Machine Learning Software. RDKIT. ORG 232. 2013. https://www.rdkit.org

[btaf326-B32] Li H , Li T, Quang D et al Network propagation predicts drug synergy in cancers. Cancer Res 2018;78:5446–57. 30054332 10.1158/0008-5472.CAN-18-0740

[btaf326-B33] Li Q , Cheng T, Wang Y et al Pubchem as a public resource for drug discovery. Drug Discov Today 2010;15:1052–7. 20970519 10.1016/j.drudis.2010.10.003 PMC3010383

[btaf326-B34] Li T-H , Wang C-C, Zhang L et al SNRMPACDC: computational model focused on Siamese network and random matrix projection for anticancer synergistic drug combination prediction. Brief Bioinform 2023;24:bbac503. 36418927 10.1093/bib/bbac503

[btaf326-B35] Liu H, Zhang W, Zou B et al DrugCombDB: a comprehensive database of drug combinations toward the discovery of combinatorial therapy. Nucleic Acids Res 2021;48:d871. 10.1093/nar/gkz1007 PMC7145671 31665429

[btaf326-B37] Meng F , Li F, Liu J-X et al NEXGB: a network embedding framework for anticancer drug combination prediction. Int J Mol Sci 2022;23:9838. 36077236 10.3390/ijms23179838 PMC9456392

[btaf326-B38] Mokhtari RB , Homayouni TS, Baluch N et al Combination therapy in combating cancer. Oncotarget 2017;8:38022–43. 28410237 10.18632/oncotarget.16723 PMC5514969

[btaf326-B39] Morgan HL. The generation of a unique machine description for chemical structures – a technique developed at chemical abstracts service. J Chem Doc 1965;5:107–13.

[btaf326-B40] Nelander S , Wang W, Nilsson B et al Models from experiments: combinatorial drug perturbations of cancer cells. Mol Syst Biol 2008;4:216. 18766176 10.1038/msb.2008.53 PMC2564730

[btaf326-B41] O’Neil J, Benita Y, Feldman I et al An unbiased oncology compound screen to identify novel combination strategies. Mol Cancer Ther 2016;15:1155–62. 26983881 10.1158/1535-7163.MCT-15-0843

[btaf326-B42] Paszke A, Gross S, Massa F et al PyTorch: an imperative style, high-performance deep learning library. Adv Neural Inf Process Syst 2019:32:8024–35.

[btaf326-B43] Preuer K , Lewis RPI, Hochreiter S et al DeepSynergy: predicting anti-cancer drug synergy with deep learning. Bioinformatics 2018;34:1538–46. 29253077 10.1093/bioinformatics/btx806 PMC5925774

[btaf326-B44] Quattrini L , Coviello V, Sartini S et al Dual Kit/Aur inhibitors as chemosensitizing agents for the treatment of melanoma: design, synthesis, docking studies and functional investigation. Sci Rep 2019;9:9943. 31289333 10.1038/s41598-019-46287-5 PMC6617451

[btaf326-B45] Rafiei F , Zeraati H, Abbasi K et al DeepTraSynergy: drug combinations using multimodal deep learning with transformers. Bioinformatics 2023;39:btad438. 37467066 10.1093/bioinformatics/btad438 PMC10397534

[btaf326-B46] Shi W , Jiang T, Nuciforo P et al Pathway level alterations rather than mutations in single genes predict response to HER2-targeted therapies in the neo-ALTTO trial. Ann Oncol 2017;28:128–35. 28177460 10.1093/annonc/mdw434 PMC5834036

[btaf326-B47] Szklarczyk D , Gable AL, Lyon D et al STRING v11: protein–protein association networks with increased coverage, supporting functional discovery in genome-wide experimental datasets. Nucleic Acids Res 2019;47:D607–13. 30476243 10.1093/nar/gky1131 PMC6323986

[btaf326-B48] Tate JG , Bamford S, Jubb HC et al COSMIC: the catalogue of somatic mutations in cancer. Nucleic Acids Res 2019;47:D941–7. 30371878 10.1093/nar/gky1015 PMC6323903

[btaf326-B49] Tooker P , Yen W-C, Ng S-C et al Bexarotene (LGD1069, Targretin), a selective retinoid X receptor agonist, prevents and reverses gemcitabine resistance in NSCLC cells by modulating gene amplification. Cancer Res 2007;67:4425–33. 17483357 10.1158/0008-5472.CAN-06-4495

[btaf326-B50] Tsigelny IF. Artificial intelligence in drug combination therapy. Brief Bioinform 2019;20:1434–48. 29438494 10.1093/bib/bby004

[btaf326-B51] Wang B , Zhang W, Zhang G et al Targeting mTOR signaling overcomes acquired resistance to combined BRAF and MEK inhibition in BRAF-mutant melanoma. Oncogene 2021;40:5590–9. 34304249 10.1038/s41388-021-01911-5 PMC8445818

[btaf326-B52] Wang X , Zhu H, Jiang Y et al PRODeepSyn: predicting anticancer synergistic drug combinations by embedding cell lines with protein–protein interaction network. Brief Bioinform 2022;23:bbab587. 35043159 10.1093/bib/bbab587 PMC8921631

[btaf326-B53] Yadav B , Wennerberg K, Aittokallio T et al Searching for drug synergy in complex dose–response landscapes using an interaction potency model. Comput Struct Biotechnol J 2015;13:504–13. 26949479 10.1016/j.csbj.2015.09.001 PMC4759128

[btaf326-B54] Yang J , Xu Z, Wu WKK et al GraphSynergy: a network-inspired deep learning model for anticancer drug combination prediction. J Am Med Inform Assoc 2021;28:2336–45. 34472609 10.1093/jamia/ocab162 PMC8510276

[btaf326-B55] Yang N , Qu Y-J, Cheng Y et al Endoplasmic reticulum stress regulates proliferation, migration and invasion of human ovarian cancer SKOV3 cells through PI3K/AKT/mTOR signaling pathway. Cancer Biomark 2017;19:263–9. 28453460 10.3233/CBM-160424 PMC13020726

[btaf326-B56] Zhao H , Zhong J, Liang X et al Application of machine learning in drug side effect prediction: databases, methods and challenges. Front Comput Sci 2025;19:195902.

[btaf326-B57] Zhao Q , Zhao H, Zheng K et al HyperAttentionDTI: improving drug-protein interaction prediction by sequence-based deep learning with attention mechanism. Bioinformatics 2022;38:655–62. 34664614 10.1093/bioinformatics/btab715

