# Supplementary material for: MADSP: predicting anti-cancer drug synergy through multi-source integration and attention-based representation learning
Source: Bioinformatics. 2025 Jun 3;41(6):btaf326. doi: 10.1093/bioinformatics/btaf326 (PMC12203510; doi:10.1093/bioinformatics/btaf326)
Supplement: btaf326_Supplementary_Data [file btaf326_supplementary_data.docx]

**MADSP: Predicting Anti-Cancer Drug Synergy**

**through Multi-Source Integration and**

**Attention-Based Representation Learning**

**(Supplementary Data)**

Yuqi Hong^1^, Qichang Zhao ^1^, and Jianxin Wang^1*^

^1^Hunan Provincial Key Lab on Bioinformatics, School of Computer Science and Engineering, Central South University, Changsha,410083, China.

* Contact: [jxwang@mail.csu.edu.cn](mailto:jxwang@mail.csu.edu.cn)

1. Classification model setup

We define drug-drug-cell line samples with synergy scores greater than 30 as positive samples and all other samples as negative samples to generate a binary classification task on the O’Neil dataset. Furthermore, the output layer of each model is added a Sigmoid activation function, which outputs the probability that a sample belongs to the positive class. Additionally, the loss function is changed from the mean squared error, to Binary Cross-Entropy loss, expressed by the following formula:

$Loss_{bce}(y_{i},{\overset{^}{y}}_{i})=-\frac{1}{N}\sum_{i=1}^{N} [y_{i}\log({\overset{^}{y}}_{i})+(1-y_{i})\log(1-{\overset{^}{y}}_{i})]$,

where $y_{i}$ is the true label of the sample (0 or 1), ${\overset{^}{y}}_{i}$ is the probability value predicted by the model, and N is the number of samples.

1. The details of all baselines

We compare our method with two classic machine learning methods and four start-of-the-art baselines:

- **Random Forest**: Random forests enable efficient regression modelling of continuous numerical outputs by constructing multiple decision trees and integrating their predictions.
- **SVR**: Support Vector Machine Regression is a supervised learning method for predicting continuous numerical outputs by finding the optimal hyperplane such that the fitting error from sample points to the hyperplane is minimised.
- **DeepSynergy**: DeepSynergy uses chemical and genomic feature vectors as inputs to predict the synergy score of drug combinations using a deep neural network model.
- **Matchmaker**: MatchMaker trains two parallel sub-networks to learn drug-specific representations on cell lines, and uses the chemical descriptors of drugs and the gene expression profiles of cell lines to predict the synergy scores of drug combinations.
- **PRODeepSyn**: PRODeepSyn integrates the PPI network and multi-omics data, utilizes Graph Convolutional Networks to optimize feature extraction for cell lines, and then combines drug molecular descriptors to perform synergy prediction.
- **SNRMPACDC:** SNRMPACDC combines Siamese network and stochastic matrix projection to predict the synergistic effects of drug combinations, improving prediction accuracy and biological interpretability through feature fusion and multi-layer perceptron networks.
- **PermuteDDS**: PermuteDDS takes a drug pair of a certain fingerprint and a cell line pair including its gene expression and gene mutation profiles as inputs, outputs different synergy profiles through three fingerprint specific networks (FSNs) and integrates them to generate a final synergy score.
- **HypertranSynergy**: HypertranSynergy uses hypergraphs to integrate relationships between cancer cell lines and drug combinations, calculate the relative importance of information at the granular level, and use the embedded obtained for drug synergy prediction.

1. Drug synergy prediction dataset

For the regression task, we used the O'Neill dataset published by Merck et al. The dataset contains 583 different combinations constructed from 38 different cancer drugs and 39 human cancer cell lines. Each experimental sample consisted of two drugs and one cell line, for a total of 22737 samples. To assess the ability to predict new drug combinations, we used five-fold cross-validation, where the dataset is divided into five unconnected folds and the same drug combination only appears in the same fold. The synergistic effects of drug combinations were expressed in four types: Loewe, Bliss, ZIP and HSA. A higher synergy score generally indicates a stronger combination synergy.

For the classification task, we used the DrugCombDB dataset. DrugCombDB contains 448555 drug-drug combinations, covering 2887 unique drugs and 124 human cancer cell lines. As we required drug structure and cell line gene expression as features, we screened these data so that drugs had structural information available from the Drugbank database and cell lines had gene expression data available from CCLE. At the same time, there were some replicates in the original experiment, and for each triplet we averaged the collaborative scores of the replicates. The final reduced dataset consisted of 69,436 samples, with 764 unique drugs and 76 unique cell lines. For the DrugCombDB dataset, synergy scores are expressed as ZIP values. Since the synergy score is a continuous value, we set the threshold to 0 to separate the data sample into synergy and antagonism.

**Table S1.** Drug synergy prediction dataset.

| Dataset | Sample | Drug | Cell |
| --- | --- | --- | --- |
| O'Neil | 22,737 | 38 | 39 |
| DrugCombDB | 448,555 | 2,887 | 124 |

**Table S2.** Hyperparameter setting.

| Hyperparameter | Space | Value |
| --- | --- | --- |
| Hidden units | [8192, 4096]; [4096, 2048]; [2048, 1024];  [8192, 4096, 2048]; [4096, 2048, 1024];  [2048, 1024, 512]; | [2048, 1024, 512]; |
| Hidden dim of Autoencoder | 512, 768, 1024 | 768 |
| Attention heads | 4, 8, 12 | 8 |
| Learning rate | 10^-5^, 10^-4^, 10^-3^, 10^-2^ | 10^-4^ |
| Dropout | No dropout; 0.1; 0.2; 0.3; 0.4; 0.5 | 0.2 |

1. Hyperparameter setting

We systematically explored various hyperparameter settings, including the number and architecture of neurons in the prediction module, the dimension of Autoencoder, and the number of heads in the multi-head attention mechanisms. **Table S2** summarizes the hyperparameter space investigated and the optimum values. For predicting the number of hidden layers in the module, we evaluated configurations with either two or three hidden layers, where the neuron count in the first hidden layer was set to 2048, 4096, or 8192, respectively. The neuron count in subsequent hidden layers was halved sequentially. Hidden units denote the number of neurons in each layer of the network in the prediction module, e.g. [8192, 4096] means that there are 8192 neurons in the first layer and 4096 neurons in the second layer.

1. Performance evaluation of classification task on the DrugCombDB dataset

To further investigate the generalization performance of the model on different datasets, we compare it with the classification model GraRep, DeepSynergy, DFFNDDS, GraphSynergy, NEXGB, and DeepTraSynergy on the DrugCombDB dataset. The experimental results are presented in Table S3.

**Table S3.** Comparison of the results of MADSP with baselines on the DrugCombDB dataset for the classification task.

| Methods | Precision | F1 | Recall | AUC | ACC |
| --- | --- | --- | --- | --- | --- |
| GraRep [1] | 0.597 | 0.613 | 0.581 | 0.728 | 0.677 |
| DeepSynergy [2] | 0.634 | 0.643 | 0.626 | 0.746 | 0.685 |
| NEXGB [3] | 0.716 | 0.729 | 0.704 | 0.833 | 0.762 |
| DFFNDDS [4] | 0.759 | 0.791 | 0.825 | 0.816 | 0.739 |
| GraphSynergy [5] | 0.723 | 0.728 | 0.718 | 0.835 | 0.755 |
| DeepTraSynergy [6] | 0.755 | 0.761 | 0.801 | 0.932 | 0.772 |
| **MADSP** | **0.798** | **0.792** | **0.814** | **0.865** | **0.776** |

MADSP achieves the best prediction results. The baselines, DeepTraSynergy and GraphSynergy, train the model in the form of composition by fusing drug features to build a drug-target association network and a protein-protein association network. Although above baselines have certain advantages in prediction accuracy, the complexity is high, and they need to be retrained when new nodes or edges are added. MADSP is not affected by this aspect and can achieve better prediction performance. DFFNDDS extracts the pre-trained features of drugs by BERT pre-training model, and combines the multi-source features of drugs by combining the molecular fingerprint features of drugs. While DFFNDDS can obtain the drug embeddings according to generalisation by combining the pre-trained features of drugs, these features are derived from the chemical structure of the drugs themselves, whereas MADSP provides a richer biological perspective by combining the information of drugs, targets and pathways.

[1] Ji B Y, You Z H, Cheng L, et al. Predicting mirna-disease association from het erogeneous information network with grarep embedding model [J]. Scientific reports, 2020, 10(1): 6658.

[2] Preuer K, Lewis R P, Hochreiter S, et al. Deepsynergy: predicting anti-cancer drug synergy with deep learning [J]. Bioinformatics, 2018, 34(9): 1538-1546.

[3] Meng F, Li F, Liu J X, et al. Nexgb: A network embedding framework for anticancer drug combination prediction [J]. International Journal of Molecular Sciences, 2022, 23(17): 9838.

[4] Xu, Mengdie, et al. "DFFNDDS: prediction of synergistic drug combinations with dual feature fusion networks." Journal of Cheminformatics 15.1 (2023): 33.

[5] Yang J, Xu Z, Wu W K K, et al. Graphsynergy: a network-inspired deep learning model for anticancer drug combination prediction [J]. Journal of the American Medical Informatics Association, 2021, 28(11): 2336-2345.

[6] Rafiei F, Zeraati H, Abbasi K, et al. Deeptrasynergy: drug combinations using multimodal deep learning with transformers [J]. Bioinformatics, 2023, 39(8): 438.

1. Attention score visualization experiment

We improve the interpretability of the model by increasing the visualization effect to show the importance of different features to the model. We take the synergistic triad [Sorafenib, Dasatinib, A2058], [Sunitinib, Lapatinib, SKOV3] as examples and visualise their drug feature attention scores. Figure S1 shows the distribution of attention weights, in which the Y-axis represents the drug name, the X-axis represents the feature, M (Morgan Fingerprint), S (Similarity), T (Target), P (Pathway). From the Figure S1, we can see that all drugs had a large weight of attention on similarity features and Morgan fingerprint features, indicating that the chemical structure of the drug was the most important part of all features. In addition, the combination of Sorafenib and Dasatinib had a synergistic effect on the melanoma cell line A2058. Sorafenib can reduce melanoma cell proliferation by inhibiting BRAF and KIT, the targets of sorafenib [1,2]. Dasatinib can induce cell apoptosis and inhibit cell invasion by regulating the corresponding MAPK/ERK pathway [3]. The attention score map shows that the target feature of Sorafenib have a higher attention score, while the pathway feature of Dasatinib get a higher attention score, which is consistent with existing research results. As for the case of [Sunitinib, Lapatinib, SKOV3], Sunitinib regulates the PI3K/AKT/mTOR pathway, and Lapatinib inhibits HER2 target protein, which can inhibit SKOV3 cell proliferation and tumor growth [4]. The target feature of Lapatinib have a higher attention score, while the pathway feature of Sunitinib get a higher attention score. The above case study illustrates that it is straightforward to observe from the attention matrix which drug information (e.g., target, pathway information, or chemical structure) has a higher weight on model predictions and may be consistent with the drug mechanism of action.


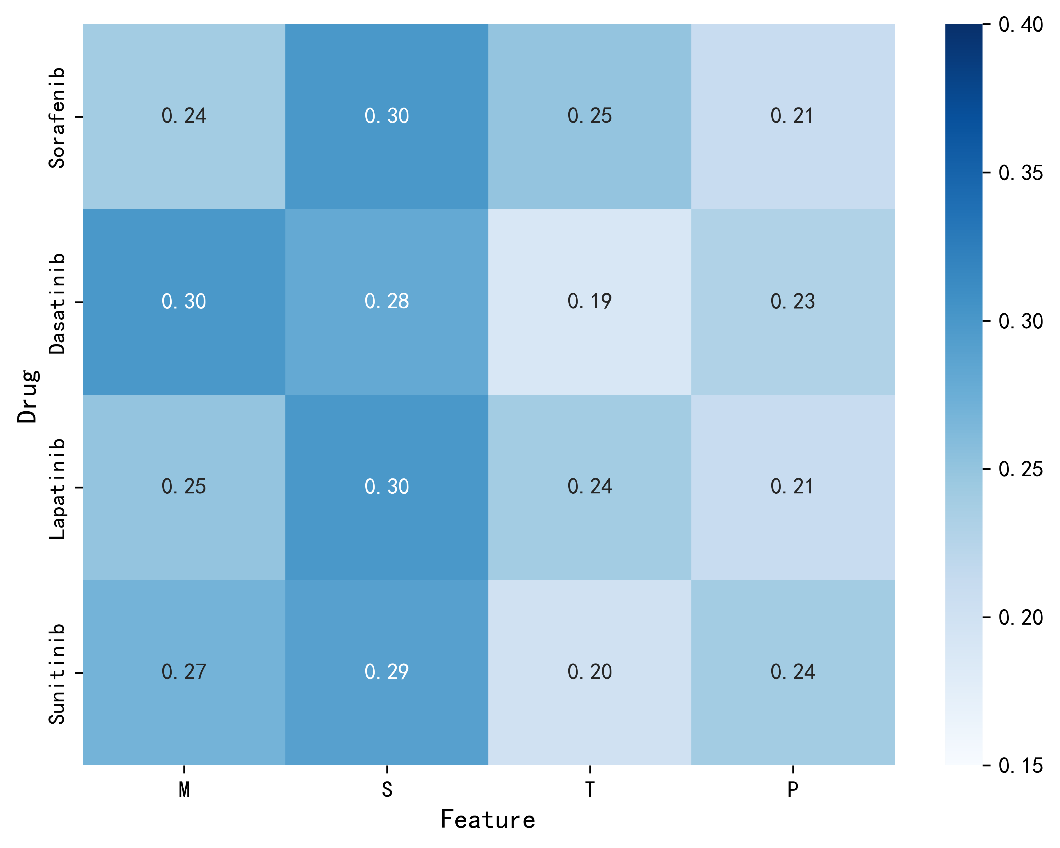


**Figure S1**. Attention score visualization of the synergistic triads [Sorafenib, Dasatinib, A2058] and [Sunitinib, Lapatinib, SKOV3].

[1] Wang B, Zhang W, Zhang G, et al. Targeting mtor signaling overcomes acquired resistance to combined braf and mek inhibition in braf-mutant melanoma [J]. Oncogene, 2021, 40(37): 5590-5599.

[2] Quattrini L, Coviello V, Sartini S, et al. Dual kit/aur inhibitors as chemosensitizing agents for the treatment of melanoma: Design, synthesis, docking studies and functional investigation [J]. Scientific reports, 2019, 9(1): 9943.

[3] Guan J, Huang H. Chrysophanol induces cell apoptosis and suppresses cell invasion by regulating akt and mapk signaling pathway in melanoma cells [J]. ScienceAsia, 2022, 48(5): 558.

[4] Yang N, Qu Y J, Cheng Y, et al. Endoplasmic reticulum stress regulates proliferation, migration and invasion of human ovarian cancer skov3 cells through pi3k/akt/mtor signaling pathway [J]. Cancer Biomarkers, 2017, 19(3): 263-269.

1. Visualization of cell line embeddings

By reducing the dimension of the cell line features from 14,091 to 768, the feature dimension of the autoencoder is significantly reduced. We use t-SNE to extract the cell line embedding vector for two-dimensional space visualization. Figure S2 shows the results of t-SNE. The color of each point represents the organization of the cell line. The embeddings of different cell lines belonging to the same tissue are clustered. The results illustrate that the autoencoder not only reduces the dimension of the cell features, but also preserves the effective information in the original features.


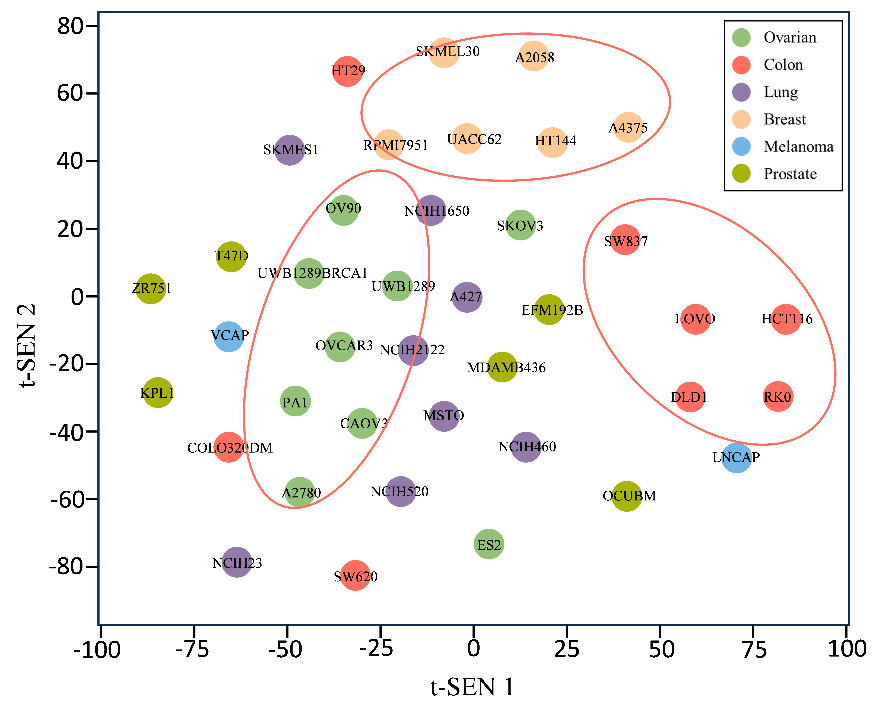


**Figure S2**. Visualization of cell line embeddings in 2D space using t-SNE

1. Ablation studies under Leave-one-drug-out setting

We design a ablation experiment on "Leave-one-drug-out" setting to emphasize the usefulness of structural similarity, target and pathway. In this experiment, the variant model Ms represents the removal of the chemical structural features, Mt represents the removal of the target features, and Mp represents the removal of the pathway features of drugs. As shown in Table S4, removing the chemical structure feature of drugs has the greatest impact on the results. The chemical structure feature contains the most characteristic information, so the current methods retain the chemical structure feature. After removing the target and pathway features of drugs, the synergy prediction ability of the drug in the new drug scenario decreased to a certain extent. Since pathway information can reveal drug interactions in biological networks and help identify potential synergistic mechanisms and target information provides a direct link between drugs and biomolecules, the introduction of drug target and pathway information can effectively improve the predictive ability of the model in the "Leave-one-drug-out" scenario.

**Table S4.** Performance evaluation of ablation studies under Leave-one-drug-out setting.

| Methods | RMSE | PCC | SCC | CI |
| --- | --- | --- | --- | --- |
| Ms | 20.49 | 0.47 | 0.46 | 0.65 |
| Mt | 19.73 | 0.49 | 0.47 | 0.67 |
| Mp | 19.54 | 0.50 | 0.49 | 0.67 |
| **MADSP** | **18.95** | **0.52** | **0.50** | **0.68** |
